# Amaranth Protein Hydrolysates Efficiently Reduce Systolic Blood Pressure in Spontaneously Hypertensive Rats

**DOI:** 10.3390/molecules22111905

**Published:** 2017-11-09

**Authors:** Giovanni Ramírez-Torres, Noé Ontiveros, Verónica Lopez-Teros, Jesús Aurelio Ibarra-Diarte, Cuauhtémoc Reyes-Moreno, Edith Oliva Cuevas-Rodríguez, Francisco Cabrera-Chávez

**Affiliations:** 1 Nutritional Sciences, Department of Chemical and Biological Sciences, University of Sonora, Hermosillo, Sonora 83000, Mexico; giovannirt2@hotmail.com (G.R.-T.); veronica.lopez@unison.mx (V.L.-T.); 2 Faculty of Physical Education and Sport, University of Sinaloa, Culiacán, Sinaloa 80019, Mexico; 3 Nutrition Sciences Academic Unit, University of Sinaloa, Culiacán, Sinaloa 80019, Mexico; aurelio_10_9@hotmail.com; 4 Faculty of Chemical and Biological Sciences, University of Sinaloa, Culiacán, Sinaloa 80199, Mexico; creyez@uas.edu.mx (C.R.-M.); edith.oliva@gmail.com (E.O.C.-R.)

**Keywords:** antihypertensive peptides, amaranth, ACE-I inhibition

## Abstract

Alcalase is the enzyme of choice to release antihypertensive peptides from amaranth proteins, but the hydrolysis conditions have not been optimized yet. Furthermore, in vivo assays are needed to confirm such a hypotensive effect. Our aim was to optimize the hydrolysis of amaranth protein with alcalase and to test in vivo the hypotensive effect of the hydrolysates. A response surface analysis was carried out to optimize the hydrolysis reaction. The response variable was the Angiotensin Converting Enzyme (ACE-I) inhibition. The hydrolysis degree was determined (free alpha-amino groups measurement). The optimized hydrolysate bioavailability was assessed in the sera of mice and the hypotensive effect was assessed in spontaneously hypertensive rats. Control groups were administered captopril or water. The optimized hydrolysis conditions were: pH = 7.01, temperature = 52 °C, enzyme concentration 0.04 mU/mg, and time = 6.16 h. The optimized hydrolysate showed a 93.5% of ACE-I inhibition and a hydrolysis degree of 74.77%. After supplementation, the hydrolysate was bioavailable in mice from 5 to 60 min, and the hypotensive effect started at 4 h in spontaneously hypertensive rats (*p* < 0.05 vs. water group). This effect was similar to the captopril hypotensive effect for the next 3 h (*p* > 0.05). The use of amaranth-optimized hydrolysates as hypotensive supplements or ingredient for functional foods seems feasible.

## 1. Introduction

Hypertension is a risk factor for developing cardiovascular diseases such as coronary heart disease, atrial fibrillation, and heart failure, among others [1,2]. Some food proteins contain peptide sequences with different functions, including antihypertensive capacity [3,4]. Amaranth (Amaranthus spp.) protein contains such bioactive peptides. More than 60 amaranth antihypertensive peptides have been identified to date [5]. Different enzymes have been used to hydrolyse amaranth proteins, but the catalysis by alcalase (E.C. 3.4.21.62) releases the peptides with the highest capacity to inhibit the angiotensin-I-converting enzyme (ACE-I) (E.C. 3.4.15.1) [6]. ACE-I is involved in the vasoconstriction process and its inhibition in in vitro assays is a common approach in the search for molecules with potential antihypertensive activity [7,8]. Due to its practicability, the in vitro ACE-I assay is more frequently performed than the evaluation of the in vivo hypotensive effect of peptides. However, in vitro assays do not always resemble in vivo assays. If the amaranth antihypertensive peptides are to be used as a dietary supplement or ingredient for the development of functional foods, their effectiveness in vivo should be proven. Thus, our aim was to optimize the hydrolysis with alcalase of a commercially available amaranth protein isolate and to evaluate in vivo the hypotensive effect of the optimized hydrolysate.

## 2. Results

### 2.1. Protein Extraction

Protein was extracted/concentrated from a commercially available amaranth protein isolate (COPRAM™). The final protein content was 74.1%, which is higher than that reported by Tapia-Blácido et al. (62.9–73.8%) [9] and Escudero et al. (52.56%) [10]. Notably, these authors started the protein extraction process from raw amaranth flour instead of an amaranth protein isolate with ~30% of protein content, as we did in this study.

### 2.2. Response Surface Optimization for Enzymatic Hydrolysis

The optimization of the hydrolysis reaction included 24 possible combinations of the variables (pH, reaction time, enzyme concentration, and temperature), and ACE-I inhibition was the response variable. Duplicates of all 24 reactions were evaluated and the central conditions had four replicates, accounting for a total of 52 reactions to test.

After the response surface analysis, the maximum theoretical value of ACE-I inhibition was calculated as follows:

% INHIBITIÓN = 841.24547 + 259.14341(A) − 1.29022 (B) + 607.92789 (C) + 0.10588 (D) + 0.073108 (AB) + 23.08195 (AC) − 0.31127 (AD) − 15.39774 (BC) − 0.045149 (BD) + 50.64199 (CD) − 18.76887 (A2) + 0.029905 (B2) − 935.75912 (C2) + 0.32099 (D2)

where: A = pH; B = temperature (°C); C = enzyme concentration (mU/mg); and D = time (h). The maximum theoretical percentage of ACE-I inhibition was 88.13%. The predicted hydrolysis reaction conditions were pH = 7.01; temperature 52 °C; enzyme concentration 0.04 mU/mg; and reaction time 6.16 h (optimized hydrolysate). The hydrolysis degree of the optimized hydrolysate was 74.77% and showed a percentage of ACE-I inhibition of 93.53%.

### 2.3. Bioavailability Test

**Figure 1:**
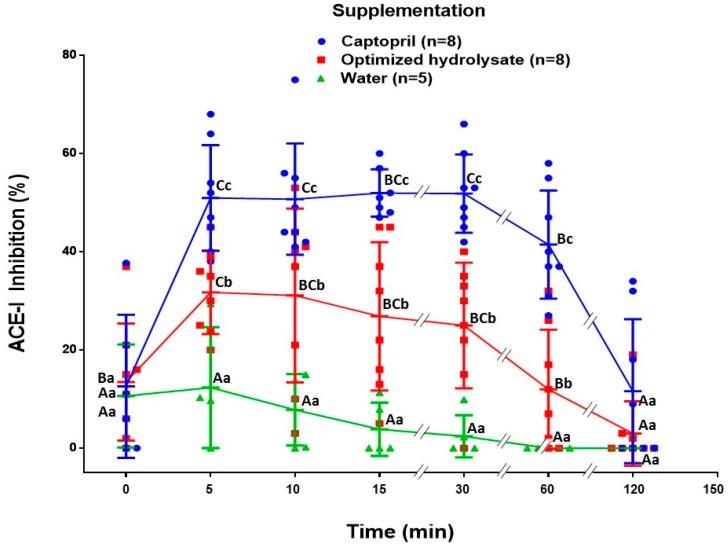
Bioavailability of antihypertensive peptides measured as a percentage of ACE-I inhibition. Sera from mice supplemented with captopril (25 mg/kg), optimized hydrolysate (2.4 gr/kg), and water (0.3 mL) were utilized for the assays. Different upper case letters mean significant differences (*p* < 0.05) in the same group (from left to right). Different lower case letters mean significant differences (*p* < 0.05) among the groups at specific time points.

The bioavailability of antihypertensive peptides was assessed in BALB/c mice. The presence of ACE-I inhibitory peptides in blood was indirectly evaluated using the ACE Inhibition kit (ACE kit-WST-Dojindo Molecular Technologies, Inc., Kumamoto, Japan). Figure 1 shows the percentages of ACE-I inhibition at different times post-supplementation with captopril, the optimized hydrolysate, and water. Five and 10 min post-supplementation, the captopril and hydrolysate groups showed the highest percentage of ACE-I inhibition (*p* < 0.05 vs. water group). Previous studies have shown that in murine models, the maximum concentration of captopril in blood is reached during the first 20 min post-supplementation [11]. Regarding the hydrolysate group, even after 60 min post-supplementation the sera from this group of mice showed a significant percentage of ACE-I inhibition compared to the water group (*p* < 0.05). As expected, the captopril group showed the highest percentage of ACE-I inhibition in a time period of 60 min (*p* < 0.05). Finally, the percentages of ACE-I inhibition were not different among groups after 120 min (*p* > 0.05).

### 2.4. Effect on Blood Pressure

**Figure 2:**
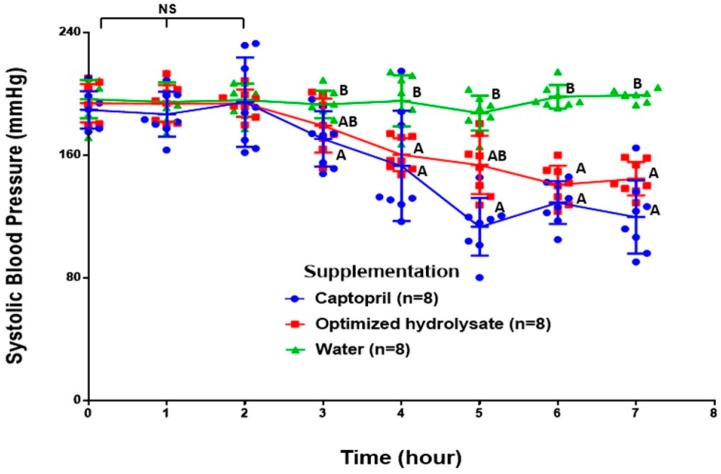
Systolic blood pressure in spontaneously hypertensive rats after supplementation with captopril (25 mg/kg body weight), optimized hydrolysate (1.2 g/kg body weight), and water (1.5 mL). Different letters mean statistical difference (*p* < 0.05). NS; non-significant difference.

Figure 2 shows the effect of the supplementation with captopril, optimized hydrolysate, and water on the systolic blood pressure of spontaneously hypertensive rats. The systolic blood pressure significantly decreased after 3 h post-supplementation with captopril, and after 4 h with the hydrolysate, compared to the water group (*p* < 0.05) (Figure 2). In the water group, the systolic blood pressure remained consistent during the time period evaluated (7 h). The lowest mean values of systolic blood pressure were reached after 5 h (110.16 mmHg) and 6 h (140.65 mmHg) post-supplementation for the captopril and hydrolysate groups, respectively. Even when the mean values of systolic blood pressure were always lower in the captopril group, there were no significant differences between the captopril and the hydrolysate supplemented groups throughout the assay (*p* > 0.05) (Figure 2).

## 3. Discussion

In this study we carried out a response surface analysis in order to establish the optimized hydrolysis conditions with alcalase to generate antihypertensive peptides from amaranth protein. Other studies have shown the antihypertensive properties of amaranth proteins [5,6,12], and a patent to obtain antihypertensive peptides from amaranth seed proteins using alcalase is also available [13]. However, only a few studies have reported the antihypertensive potential of amaranth peptides in vivo and, to our knowledge, there is scarce information about the optimized conditions to generate these bioactive peptides. Here, we present an optimized amaranth protein hydrolysate, which could have a higher potential to inhibit ACE-I in vitro than that reported by others [6], and this could be attributed to the optimized process of hydrolysis with alcalase based on a response surface analysis. Other enzymes (e.g., trypsin) have been used to hydrolyse amaranth protein in order to obtain ACE-I inhibitory peptides [5]. However, Fritz et al. showed that amaranth hydrolysates obtained with alcalase have a higher percentage of ACE-I inhibition than those obtained with other proteolytic enzymes such as trypsin, chymotrypsin, pronase, and papain. The enzyme specificity to brake down peptide bonds is important to generate the amaranth antihypertensive peptides. For instance, trypsin has preference for positively charged Lysine/Arginine residues, while alcalase (EC 3.4.21.62) has a broad specificity for peptide bonds and preference for several uncharged residues [14]. This could increase the diversity of the peptide sequences generated after alcalase hydrolysis of amaranth proteins and be a key factor in the generation of peptides with antihypertensive capacity.

Our results suggest that the optimized amaranth hydrolysate is bioavailable and the compound/molecules that reach the blood stream have the potential to inhibit ACE-I in vitro. This effect can be detected after 5 min of the intragastric administration of the optimized hydrolysate. Previous studies have reported that antihypertensive peptides can be detected in blood after 5 min post-oral-challenge and reach a maximum level after 30 min of administration [15]. Notably, sera from the hydrolysate group obtained after 60 min post-oral-challenge were able to inhibit ACE-I in vitro. This is of relevance, as only peptides with increased resistance to plasma proteases could remain in the blood stream at such a time [16]. Antihypertensive peptides labile to plasma proteases are almost so completely degraded after 30 min that they have reached the blood stream [16]. Similarly, the sera from the captopril group inhibited the ACE-I from 5 min and up to 60 min post-supplementation. This finding is in line with other studies of bioavailability of captopril carried out in murine models [11].

Compared to the time points from 10 min to 120 min, the sera from the water group (negative control) collected at the time points 0 min and 5 min showed a slightly potential to inhibit ACE-I. This behavior could be attributed to the presence of enkephalins. These compounds are released after a stressing episode such as the intragastric administration of the treatments [17]. Enkephalins are ACE-I substrates and/or inhibitors [18], but they are rapidly metabolized and consequently have a very short half-life.

Bioactive compounds are resistant to gastrointestinal digestion, are bioavailable, are physiologically relevant, and have potential to be used as supplements or ingredients for functional foods development. The optimized amaranth hydrolysate efficiently reduced the systolic blood pressure in spontaneously hypertensive rats, 4 h post-intragastric administration. Notably, the administered doses of the optimized hydrolysate were half of the doses previously reported (1.2 vs. 2.4 g/kg) [5], and this was enough to trigger a hypotensive effect for at least three hours. Unfortunately, our study did not evaluate the systolic blood pressure in spontaneously hypertensive rats later than 7 h after the treatments were administered. Fritz et al. [6] reported a maximum hypotensive effect of an alcalase digested amaranth hydrolysate after 1.5 h of administration by gastrostomy. On the contrary, when the hydrolysate was orally administered (by using feeding tubes), a significant effect was seen after 3 h. Similarly, Matsui et al. [19] reported that an antihypertensive peptide and captopril could decrease the systolic blood pressure in spontaneous hypertensive rats after 3 h of oral administration (by using feeding tubes). Others have reported that the highest antihypertensive effect of the antihypertensive peptide GAAGGAF from *Coix lachryma-jobi* occurs after 4 h to 6 h of administration, and the effect remains until 7 h post-supplementation [20]. Our findings are in line with the previous studies and confirm that an optimized amaranth hydrolysate has a hypotensive effect in vivo. More importantly, this effect can be sustained for at least 3 h in spontaneously hypertensive rats. The mechanism responsible for the sustained antihypertensive effect seen in spontaneously hypertensive rats remains unknown, but it should be related to the presence of ACE-I inhibitory peptides with increased resistance to physiologically relevant proteases.

It should be acknowledged that our study has some limitations. First, it remains uncertain if the antihypertensive potential shown by the optimized hydrolysate was consequence of the release of new antihypertensive peptides with enhanced activity or the optimization of the hydrolysis reaction of amaranth protein with alcalase yielded larger numbers of already-known, antihypertensive peptides. The characterization of antihypertensive peptides, such as VPP and IPP from cow’s milk, is a key point for their use as supplements [21]. Secondly, neither the hypotensive effect of doses of amaranth hydrolysate below 1.2 g/kg nor the potential adverse effects of the regular consumption of amaranth hydrolysates were evaluated. Future studies addressing these points are warranted. Moreover, the synergistic hypotensive effect of amaranth hydrolysates consumption along with physical activity is a topic that deserves further research.

## 4. Materials and Methods

### 4.1. Extraction and Concentration of Protein

The protein concentrate was obtained from a commercially available amaranth protein isolate (COPRAM™) following the method described by Tapia-Blácido et al. [9]. The isolate (~30% protein, determined by the Kjeldahl method) was suspended in acetone (1:2 weight/volume), stirred vigorously, and centrifuged (6000× *g*, 30 min, 4 °C). The precipitate was collected and re-suspended in NaOH (62.5 mM), stirred (24 h, 4 °C), and centrifuged (6000 × *g*, 20 min, 10 °C). The supernatant was collected and the pH adjusted to 5.0 to precipitate the proteins. The suspension was centrifuged (6000× *g*, 20 min, 10 °C) and the precipitate collected and re-suspended in water, and the pH was adjusted to 7.0. Finally, the protein concentrate was freeze-dried and stored at room temperature. The total protein content was determined following the AOAC method 979.09 [22].

### 4.2. Optimization of Hydrolysis with Alcalase

A worksheet (Supplementary Table S1) was generated by using Design-Expert software 7.0.0 (Stat-Ease, Inc., Minneapolis, MN, USA) to perform response surface analysis with randomly selected reaction conditions. The evaluated conditions were enzyme concentration, temperature, reaction time, and pH. The values of the central conditions were those reported by Fritz et al. [6]. The percentage of ACE-I inhibition was the response variable and was determined by using the ACE Inhibition kit (ACE kit-WST-Dojindo Molecular Technologies, Inc.) according to the manufacturer’s instructions. The hydrolysis reactions were carried out in 1 mL vials in a Thermomixer (Eppendorf™, Hamburg, Germany). The reactions were stopped by heating the samples (85 °C, 10 min).

### 4.3. Degree of Hydrolysis

The hydrolysis degree determinations were carried out in the samples with the highest percentage of ACE-I inhibition. The quantification of free alpha-amino groups was carried out with the Trinitrobenzene Sulfonic Acid method (TNBSA) [23]. Cysteine solutions were prepared to build a standard curve (from 0 to 1.66 mMol/L). The hydrolysis degree was calculated by using the following equation: DH = (h/8.12) × 100, where DH is the hydrolysis degree expressed as a percentage; h is the concentration of free amino acids in the sample (mmol/L); and 8.12 is a constant assigned to the amaranth protein [24].

### 4.4. Animals

Female Balb/c mice (12 weeks old, 25–30 g) were provided by BioInvert (México city, México) and were used for the bioavailability assays purposes. Male spontaneously hypertensive rats (8 weeks old, 300–350 g) were provided by the Cell Physiology Institute of the National Autonomous University of Mexico (Instituto de Fisiología Celular-UNAM) and were used in the anti-hypertensive assays. An Ethics Review Board of the University of Sinaloa approved the study protocol (CE-UACNYG-2015-SEP-001). Rats and mice were placed in plastic cages with stainless steel lids. Room temperature was maintained at 28 °C with cycles of 12:12 h light-dark. Food (Rodent Lab Chow 5001) and water were available *ad libitum*.

### 4.5. Bioavailability Assay

21 Balb/c mice were divided into 3 groups. The first group (*n* = 8) received the optimized hydrolysate (2.4 g/kg of body weight) re-suspended in water [5]. The second group (*n* = 8) received the ACE-I inhibitor Captopril (25 mg/kg of body weight) solubilized in water [25]. The third group (*n* = 5) received only water (300 μL).

All treatments were administered intragastrically by using plastic feeding tubes (20 GA × 38 mm, Instech Laboratories, Inc). Blood samples were drawn from the tail vein before and after (5, 10, 15, 30, and 60 min) the intragastric interventions. The blood samples were centrifuged at 2000× *g*, 24 °C, 20 min, and the sera were collected. The presence of antihypertensive compounds in the serum samples was evaluated by using the ACE Inhibition kit (ACE kit-WST-Dojindo Molecular Technologies, inc., Kumamoto, Japan) according to the manufacturer’s instructions.

### 4.6. Effect of Supplementation on Blood Pressure

Three groups of spontaneously hypertensive rats (*n* = 8 each group) were used to monitor blood pressure after intragastric interventions. The first group received the optimized hydrolysate (1.2 g/kg of body weight); this is half of the dose of amaranth hydrolysates previously reported [5]. The second group received Captopril (25 mg/kg of body weight) solubilized in water [25,26,27]. The third group received only water. The interventions were carried out by using plastic feeding tubes (18GA × 75 mm, Instech Laboratories, Inc.). Blood pressure was measured at 0, 1, 2, 3, 4, 5, 6, and 7 h after supplementation with a CODA tail cuff blood pressure monitor (Kent Scientific, Torrington, CT, USA).

### 4.7. Statistical Analysis

A response surface analysis was carried out using Design-Expert software 7.0.0 (Stat-Ease, Inc., Minneapolis, MN, USA). The analyses of variance were carried out using the GraphPad Prism 3.0 software (Kruskal-Wallis and Dunn test). Differences were considered statistically significant at *p* < 0.05.

## 5. Conclusions

The optimized conditions for hydrolyse amaranth protein with alcalase to achieve the highest ACE-I inhibition capacity were established (pH = 7.01, T = 52.0 °C, alcalase concentration 0.04 mU/mg, and time 6.16). The optimized hydrolysate showed a degree of hydrolysis of 74.77% and a percentage of ACE-I inhibition of 93.53%. The hydrolysate was bioavailable in mice from 5 min to 60 min, similar to captopril, and started a hypotensive effect 4 h post-supplementation in spontaneously hypertensive rats. This hypotensive effect was sustained for at least 3 h and showed a trend similar to captopril. Overall, the results suggest that the use of amaranth hydrolysates as a supplement or ingredient for the development of functional foods is feasible.

## Supplementary Materials

Supplementary Materials are available online, Table S1: Response Surface Analysis.
