# Supplementary material for: Amaranth Protein Hydrolysates Efficiently Reduce Systolic Blood Pressure in Spontaneously Hypertensive Rats
Source: Molecules. 2017 Nov 9;22(11):1905. doi: 10.3390/molecules22111905 (PMC6150404; doi:10.3390/molecules22111905)
Supplement: Supplementary file 1 [file molecules-22-01905-s001.pdf]

Table S1. Response Surface Analysis. Worksheet .

Order	Order Std.	Central points	Blocks	A*	B*	C*	D*
1	43	1	2	6	5	0.04	48
2	51	0	2	7	5	0.04	48
3	49	1	2	7	5	0.04	36
4	33	1	2	6	8	0.06	36
5	48	1	2	7	5	0.06	48
6	36	1	2	8	2	0.02	60
7	40	1	2	8	2	0.06	60
8	39	1	2	6	2	0.06	60
9	32	1	2	8	2	0.06	36
10	34	1	2	8	8	0.06	36
11	30	1	2	8	8	0.02	36
12	27	1	2	6	2	0.02	36
13	50	1	2	7	5	0.04	60
14	52	0	2	7	5	0.04	48
15	45	1	2	7	2	0.04	48
16	31	1	2	6	2	0.06	36
17	38	1	2	8	8	0.02	60
18	37	1	2	6	8	0.02	60
19	42	1	2	8	8	0.06	60
20	44	1	2	8	5	0.04	48
21	41	1	2	6	8	0.06	60
22	29	1	2	6	8	0.02	36
23	46	1	2	7	8	0.04	48
24	35	1	2	6	2	0.02	60
25	47	1	2	7	5	0.02	48
26	28	1	2	8	2	0.02	36
27	5	1	1	6	2	0.06	36
28	14	1	1	8	2	0.06	60
29	10	1	1	8	2	0.02	60
30	3	1	1	6	8	0.02	36
31	4	1	1	8	8	0.02	36
32	6	1	1	8	2	0.06	36
33	21	1	1	7	5	0.02	48
34	2	1	1	8	2	0.02	36
35	12	1	1	8	8	0.02	60
36	20	1	1	7	8	0.04	48
37	19	1	1	7	2	0.04	48
38	1	1	1	6	2	0.02	36
39	9	1	1	6	2	0.02	60
40	13	1	1	6	2	0.06	60
41	15	1	1	6	8	0.06	60
42	25	0	1	7	5	0.04	48
43	16	1	1	8	8	0.06	60
44	24	1	1	7	5	0.04	60
45	26	0	1	7	5	0.04	48
46	22	1	1	7	5	0.06	48
47	11	1	1	6	8	0.02	60

48	23	1	1	7	5	0.04	36
49	18	1	1	8	5	0.04	48
50	8	1	1	8	8	0.06	36
51	17	1	1	6	5	0.04	48
52	7	1	1	6	8	0.06	36

***A:pH; B: time(h); C: enzyme concentration (mU/mg of protein); D: temperature (°C).**
